# Fusion InPipe, an integrative pipeline for gene fusion detection from RNA-seq data in acute pediatric leukemia

**DOI:** 10.3389/fmolb.2023.1141310

**Published:** 2023-06-09

**Authors:** Clara Vicente-Garcés, Joan Maynou, Guerau Fernández, Elena Esperanza-Cebollada, Montserrat Torrebadell, Albert Català, Susana Rives, Mireia Camós, Nerea Vega-García

**Affiliations:** ^1^ Pediatric Cancer Center Barcelona (PCCB), Institut de Recerca Sant Joan de Déu, Leukemia and Pediatric Hematology Disorders, Developmental Tumors Biology Group, Esplugues de Llobregat, Spain; ^2^ Hospital Sant Joan de Déu Barcelona, Genetics Medicine Section, Esplugues de Llobregat, Spain; ^3^ Institut de Recerca Hospital Sant Joan de Déu, Neurogenetics and Molecular Medicine, Esplugues de Llobregat, Spain; ^4^ Hospital Sant Joan de Déu Barcelona, Hematology Laboratory, Esplugues de Llobregat, Spain; ^5^ Instituto de Salud Carlos III, Centro de Investigación Biomédica en Red De Enfermedades Raras (CIBERER), Madrid, Spain; ^6^ Pediatric Cancer Center Barcelona (PCCB), Hospital Sant Joan De Déu Barcelona, Leukemia and Lymphoma Unit, Barcelona, Spain

**Keywords:** RNA-sequencing, bioinformatic pipelines, fusion detection, pediatric acute leukemia, molecular diagnostics

## Abstract

RNA sequencing (RNA-seq) is a reliable tool for detecting gene fusions in acute leukemia. Multiple bioinformatics pipelines have been developed to analyze RNA-seq data, but an agreed gold standard has not been established. This study aimed to compare the applicability of 5 fusion calling pipelines (Arriba, deFuse, CICERO, FusionCatcher, and STAR-Fusion), as well as to define and develop an integrative bioinformatics pipeline (Fusion InPipe) to detect clinically relevant gene fusions in acute pediatric leukemia. We analyzed RNA-seq data by each pipeline individually and by Fusion InPipe. Each algorithm individually called most of the fusions with similar sensitivity and precision. However, not all rearrangements were called, suggesting that choosing a single pipeline might cause missing important fusions. To improve this, we integrated the results of the five algorithms in just one pipeline, Fusion InPipe, comparing the output from the agreement of 5/5, 4/5, and 3/5 algorithms. The maximum sensitivity was achieved with the agreement of 3/5 algorithms, with a global sensitivity of 95%, achieving a 100% in patients’ data. Furthermore, we showed the necessity of filtering steps to reduce the false positive detection rate. Here, we demonstrate that Fusion InPipe is an excellent tool for fusion detection in pediatric acute leukemia with the best performance when selecting those fusions called by at least 3/5 pipelines.

## 1 Introduction

Gene fusions play an important role in different cancer types. These events can result from different structural variations, such as translocations, inversions, or deletions. Consequently, two separated genes will be juxtaposed, producing an aberrant protein product or a dysregulated transcription of these genes. When the affected genes are oncogenes or tumor suppressor genes, this fusion may lead to cancer development. Thus, their identification is crucial, as they can be useful for precise diagnosis, risk stratification, or as a therapy target (Mitelman et al., 2007; Gao et al., 2018).

Acute leukemia (AL) is the most common cancer type and one of the leading causes of cancer-related death in children (Hunger and Mullighan, 2015). Genetics has proved to be a powerful tool in understanding leukemia while providing essential diagnostic and prognostic information. Currently, the identification of genetic abnormalities in AL patients is performed by conventional cytogenetics, Fluorescence *In Situ* Hybridization (FISH), and retro-transcriptase (quantitative) PCR (RT-(q)PCR) (Malard and Mohty, 2020). Unfortunately, with the use of these conventional methodologies, there is a proportion of patients in whom we cannot identify genetic abnormalities (Inaba and Mullighan, 2020). Advances in Next-Generation Sequencing (NGS) have improved their identification, and NGS-targeted panels are starting to be introduced to clinical routine, facilitating the detection of less common rearrangements. However, they look into a selection of genes, and more extensive and complete studies, such as whole genome sequencing (WGS) or whole transcriptome sequencing (WTS), need to be performed to allow the detection of all the possible disease-causing alterations.

Over the last few years, RNA sequencing (RNA-seq) has become a well-established tool for the massive detection of fusion transcripts and determining gene expression profiles (LaHaye et al., 2021). Its introduction in clinical diagnostics has allowed the identification of new driver genomic lesions, which have improved the risk-stratification of the patients and have led to the establishment of new leukemia subtypes (Zaliova et al., 2019).

Although a large number of bioinformatics tools have been developed to analyze RNA-seq data to detect fusion genes, there is still no gold standard (Liu et al., 2015; Kumar et al., 2016; Haas et al., 2019). Each tool has its own configuration parameters and performance characteristics that give rise to differences in specificity and sensitivity between methods. Thus, it is crucial the selection of the bioinformatics tools that are best suited to the study being conducted (Audoux et al., 2017).

The main problem when analyzing RNA-seq data is the elevated rate of false-positive fusions detected. To increase specificity, all bioinformatic tools apply different filters in the last steps of the analysis. Sometimes these filters can be too strict; consequently, true positive fusions can occasionally be discarded. Therefore, an exhaustive evaluation of fusion calls and filtering strategies are needed to obtain good sensitivity and specificity. Furthermore, using more than one algorithm is highly recommended to obtain better accuracy when identifying gene fusion candidates (Carrara et al., 2013; Dupain et al., 2017).

Taken together, fusion gene identification has become an important part of the diagnosis in AL, and so far, different available pipelines have been used for the study of leukemia, but no unique software is able to detect all fusion alterations consistently. Thus, we continue facing different challenges when trying to detect fusion genes. To address this topic, here we present the study of the individual performance of five different pipelines and the introduction of an integrative algorithm selecting a new combination of pipelines to facilitate the analysis in clinical routine. Furthermore, we show an analysis of its performance in detecting driver fusion genes involved in childhood leukemia.

## 2 Materials and methods

### 2.1 Training datasets

We used two different datasets, public transcriptomics’ data from different cell lines and internally generated patient transcriptomic data, for the comparison of the selected pipelines and the evaluation of our integrative bioinformatics pipeline. We used cell lines data from different online repositories to determine whether the individual bioinformatics pipelines and the new integrative pipeline were useful for detecting driver, clinically meaningful leukemia rearrangements already identified by other methods. Later, all these pipelines were used to analyze our training patients’ dataset.

#### 2.1.1 Cell lines’ data

To evaluate the performance of the analysis of the bioinformatics pipelines, we used different cell lines RNA-Seq datasets deposited in public repositories (ENA number: PRJEB30312; ArrayExpress ID: E-MTAB-7721). These cell lines present a thorough genomic characterization as they have been used for years as leukemia disease models for different experiments. They have been studied cytogenetically, immunologically, and molecularly and validated by high throughput NGS approaches (whole exome sequencing and RNA-sequencing) (Quentmeier et al., 2019). We selected 14 different leukemia cell lines harboring common fusion genes in main AL subtypes. The cell lines and their genetic characteristics are listed in the (Supplementary Table S1).

#### 2.1.2 Patients’ data

A subgroup of fifteen patients harboring some of the most common leukemia rearrangements were selected and studied by RNA-seq. All the patients were previously characterized in our center by karyotype, FISH, RT-qPCR, and NGS (amplicon-based targeted panel). Supplementary Table S2 shows the main genetic characteristics of the sequenced patients.

### 2.2 RNA-seq library preparation and sequencing

Libraries were performed in our center following the TruSeq stranded mRNA-seq protocol (Illumina) and sequenced in a NextSeq500 instrument (Illumina) according to the manufacturer’s instructions. Data were analyzed using the selected bioinformatics pipelines (see below).

### 2.3 Fusion detection pipelines

The pipelines Arriba (Uhrig et al., 2021), CICERO (Tian et al., 2020), deFuse (McPherson et al., 2011), FusionCatcher (Nicorici et al., 2014)and STAR-Fusion (Haas et al., 2017) were selected due to having good-performance and as the representation of a wide spectrum of fusion calling strategies based on literature search. All the software packages were downloaded, installed, and run using the default configuration on our server. A brief description of the selected pipelines is given in the following sections.

#### 2.3.1 Arriba (AR)

Command-line tool developed for gene fusion detection from pair-end and single-end RNA-seq data in clinical research. It also detects other structural rearrangements, such as internal tandem duplications (ITD), etc. It uses STAR aligner to align sequences and obtain the list of fusion candidates. STAR searches for split reads and spanning reads in order to detect chimeric alignments.

#### 2.3.2 CICERO (CI)

Local assembly-based algorithm set up for fusion gene detection as well as ITDs. STAR is used for the alignment and mapping followed by duplicate reads removal with Picard. The candidate fusion list is generated by the identification of soft-clipped reads. Then, fusion contigs are assembled and mapped, using BLAT to identify the breakpoint.

#### 2.3.3 DeFuse (DF)

This algorithm was developed to detect gene fusions from RNA-seq data. It uses discordant paired-end alignments to identify split reads, analyze, and find fusion boundaries. Predicted fusions are then filtered to reduce the false positive candidates. Finally, a list of fully annotated predicted fusions is generated.

#### 2.3.4 FusionCatcher (FC)

This software tool was generated to detect fusion genes in pair-end RNA-seq data from vertebrates annotated in the Ensembl database. It performs a quality filtering of the reads before starting with the alignment. Mapped reads are used to generate a preliminary list of candidates by searching spanning reads. To determine the exact breakpoint, it uses a combination of four aligners, Bowtie, BLAT, STAR, and Bowtie2.

#### 2.3.5 STAR-fusion (SF)

This bioinformatic tool uses the STAR aligner to align reads from RNA-seq data, ideally, pair-end reads, and identify candidate fusion transcripts. The output generated is processed by STAR-fusion to map split and spanning reads to a reference annotation set.

### 2.4 Filtering steps

Different filters were manually applied to the candidate list of called fusion genes in order to reduce the number of artifacts and identify those fusions with potential clinical relevance in leukemia, considering the current knowledge. Each of the pipelines has different output files depending on their internal analysis workflow and consequently they display different information of the called fusion genes (Supplementary Table S3; Supplementary Table S4). However, most of the given information is similar for all the pipelines and therefore the filters that were applied were all in the same direction; reduce and eliminate artifacts and FP variants. To achieve this, we focused on discarding those calls with low confidence, a low number of reads evidencing the presence of a fusion (split and spanning reads) and, if available, we considered their pathogenicity and other information of interest such as their presence in healthy cohorts or information of the genes involved in the fusion. Filters applied to each pipeline are listed in the Supplemental Material.

Additionally, a manual inspection of the remaining fusion candidates was performed. First, read-through fusions (e.g., MTAP:CDKN2B-AS1 or RAG1:IFTAP), out-of-frame fusions, and those fusions including breakpoints out of splice-sites or coding exons were discarded. Fusion genes containing repeat regions, paralogs, pseudogenes, genes rearranged with more than one gene in the same patient (promiscuous genes), and rearrangements involving genes not related to leukemia were also removed. Those fusions containing genes with clinical relevance in leukemia were retained on the candidate list, regardless its promiscuity or nature of the rearrangement, to determine later their possible impact on the disease. Secondly, final candidate rearrangements were visually inspected using the Integrative Genomics Viewer (IGV). By introducing the breakpoint coordinates on the tool, a split screen is opened, and we can visualize the alignments. When a fusion gene is formed, part of the reads are aligned correctly on the gene but the other part can be seen erroneously aligned, confirming that a rearrangement has been produced. Moreover, a blast of the sequence must confirm that the non-aligned part of the read belongs to the expected partner of the gene fusion to support the fusion. Finally, a bibliographic review of the final candidates was performed to obtain information about the rearrangement in case it had been previously described and determine its possible pathogenicity or its involvement in leukemia.

### 2.5 Fusion gene integration pipeline (Fusion InPipe)

As individual fusion callers may miss some of the fusion candidates, we developed the Fusion Gene Integration Pipeline [Fusion InPipe (FIP)], based on the MetaFusion model (Apostolides et al., 2021), to standardize and integrate the outputs from various fusion callers and get a better performance than using the fusion callers individually.

**FIGURE 1:**
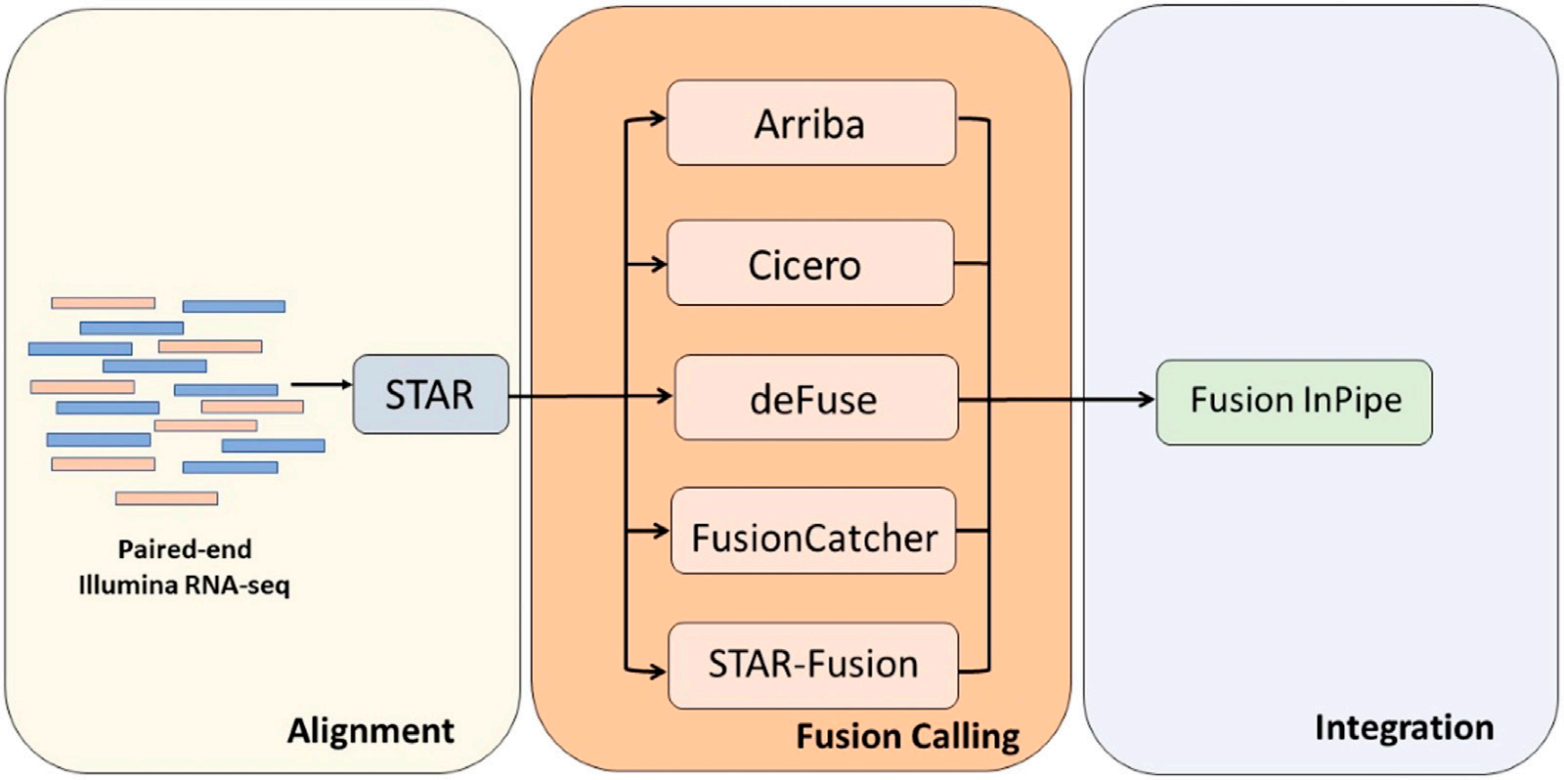
The Fusion InPipe workflow for processing RNAseq data. Fusion InPipe contains three modules: Alignment, Fusion Calling, and Integration.

Fusion InPipe has a modular pipeline architecture implemented in bash and Python-based frameworks as shown in Figure 1. In the first module, paired-end RNA-seq FASTQ files are mapped to reference genome GRCh38 using STAR aligner. Next, each caller runs independently to get its own fusion call output. Lastly, the output files generated for each caller are standardized to facilitate the combination of the results of the different callers. The final output includes one file with all the reported fusions by all the algorithms in a standard format and a summary file with the fusion callers’ information (number and names) that detect each fusion event.

For the analysis of the fusion genes called by Fusion InPipe, we first had a look at those fusions called by all the pipelines, then at the rearrangements detected by a minimum of 4 pipelines, and finally, the candidates called by at least 3 of the five pipelines. To reduce the possible false-positive variants, we applied the above-mentioned filters.

### 2.6 Accuracy assessment of the pipelines

True positive (TP), false positive (FP), and false negative (FN) fusion predictions were assessed for the different methods.

In order to ensure the performance of the tool in clinical practice, TP fusions were defined as those gene fusions with clinical impact previously reported by other methodologies in the analyzed cell lines or patients and called by the different pipelines.

FN fusions were defined as gene fusions previously reported by other methodologies that failed to be detected by RNA-seq.

Finally, in our study, we considered as FP fusions all the detected fusions by RNA-seq that were not previously reported in the comprehensively analyzed cell lines or patient samples. However, for future studies in a discovery setting, not previously described variants should not automatically be named false positive fusions and should be confirmed by other methodologies to rule out artifacts.

Sensitivity and precision values were calculated as:

$$Sensitivity=\left(TPfusions\;identified\right)/\left(\left(TP+FNfusions\right)\right)$$

$$Precision=\left(TPfusions\;identified\right)/\left(\left(TP+FPfusions\right)\right)$$

To define the general performance of the pipelines we calculated the F1-score**. F1-score** is a measure that combines sensitivity and precision. An F1-score of 0 indicates a poor measure and therefore a bad performance of the tool, while a good F1-Measure score should be near 1. F1-score is calculated with the following equation:

$$F1\;Score=2\times \left(\left(Sensitivity\times Precision\right)/\left(Sensitivity+Precision\right)\right)$$

## 3 Results

In order to evaluate if the selected pipelines were able to detect leukemic driver rearrangements, we used RNA-seq data from different leukemia cell lines (available in public repositories), and from a cohort of patients sequenced in our center.

**FIGURE 2:**
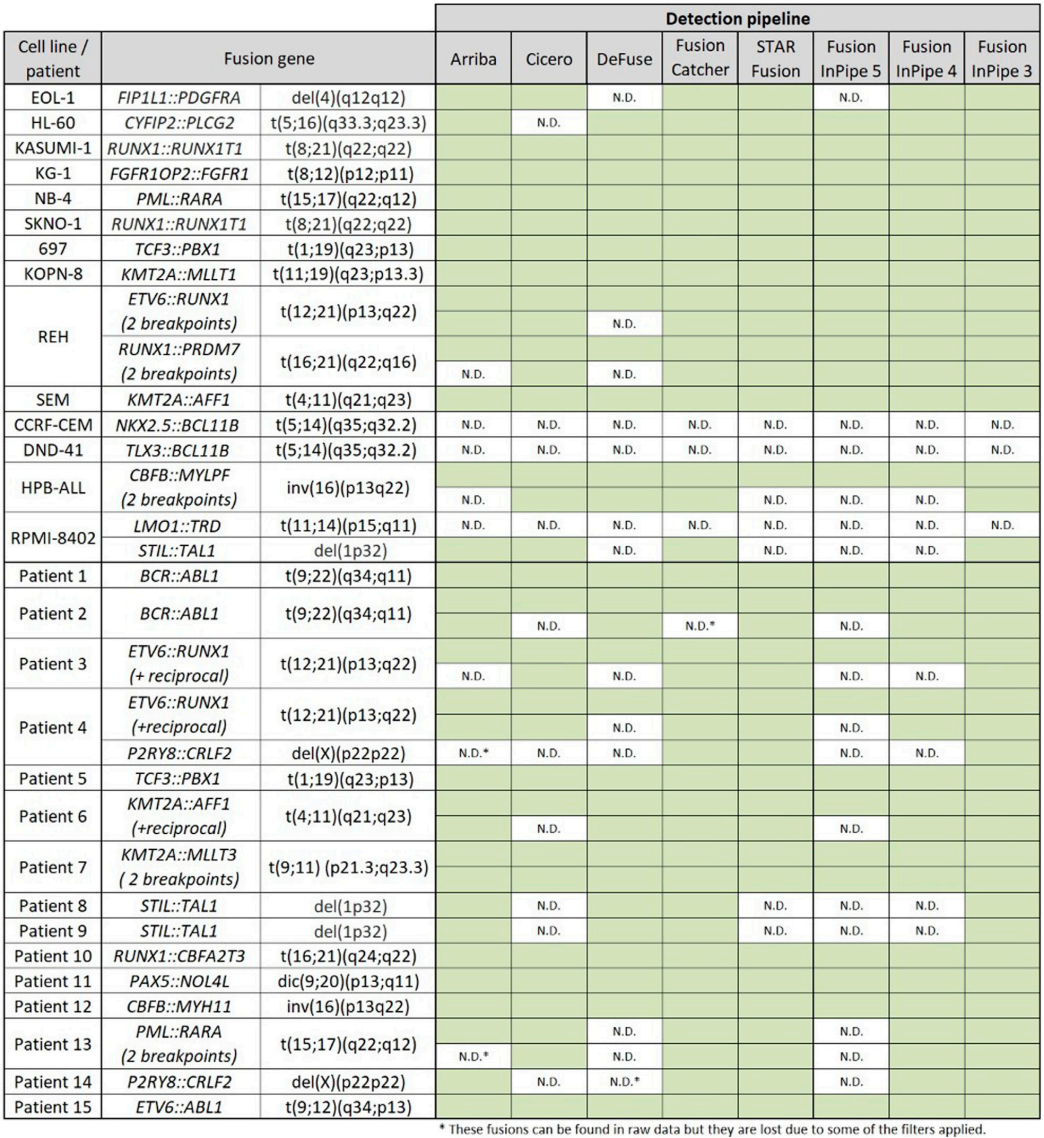
Rearrangements detected by the different pipelines. Green boxes indicate the correct detection of the alteration. N.D. indicates no detection by the algorithm.

We first assessed the performance of each algorithm separately and compared the results obtained for each sample and patient. Secondly, we performed Fusion InPipe by combining the outputs of the different pipelines in just one file. For the Fusion InPipe, we compared the outputs obtained from the agreement of 5/5, minimum 4/5, and minimum 3/5 pipelines for both cell lines and patients’ data. In global, the different algorithms separately, as well as Fusion InPipe, detected most of the expected rearrangements on the cell lines and the patients’ data (Figure 2).

### 3.1 Fusion detection by individual currently available bioinformatics pipelines

First, we analyzed the different pipelines separately. Each of the bioinformatics pipelines inspected 14 cell lines. The total number of fusions called prior to and after applying manual filters and manual inspection differed between the different tools. However, at the end of the analysis, all the pipelines detected most of the expected rearrangements.

#### 3.1.1 Fusion detection in cell lines’ data

The initial number of calls by the different pipelines was 584 (AR), 960 (CI), 6104 (DF), 7976 (FC), and 114 (SF). After manual filtering, the number of candidate fusions was drastically reduced to 37 (AR), 28 (CI), 61 (DF), 159 (FC) and 19 (SF). However, not all the artifacts were discarded at this step. Finally, a manual curation based on IGV visualization and a bibliographic review of the filtered candidates was needed to just retain the TP fusions. The final list of candidates varied from 15 to 22, depending on the pipeline.

In this dataset, all the fusions were called by more than one pipeline except for *NKX2.5::BCL11B*, *TLX3::BCL11B,* and *LMO1::TRD*, which were not detected by any of the studied pipelines. FusionCatcher was able to detect all the fusions except the ones mentioned before. Arriba, CICERO, deFuse, and STAR-fusion, failed in the identification of other rearrangements (Figure 2).

The sensitivity and precision for each pipeline are shown in Table 1 and Supplementary Figure S1. FusionCatcher achieved the highest sensitivity (84.2%) followed by Arriba (73.7%), CICERO (73.3%), and STAR-Fusion (73.7%) while deFuse had the lowest sensitivity (63.2%). Regarding precision, Arriba and CICERO obtained the highest value (93.3%), and deFuse obtained the minimum value (66.67%).

**TABLE 1:** Performance of each algorithm, individually or ensembled, in cell lines’ data, patients’ sample data, and global analysis. Total fusions and true positive fusions are referred to the number of gene fusions identified after manual curation.

Bioinformatic pipeline	Total fusions identified	True positive fusions identified	Sensitivity	Precision	F1-score
CELL LINES					
Arriba	15	14	73.7	93.3	0.82
CICERO	15	14	73.7	93.3	0.82
DeFuse	18	12	63.2	66.7	0.65
FusionCatcher	22	16	84.2	72.7	0.78
STAR-Fusion	19	14	73.7	73.7	0.74
Fusion InPipe 5 callers	14	13	68.4	92.9	0.79
Fusion InPipe 4 callers	16	14	73.7	87.5	0.80
Fusion InPipe 3 callers	20	16	84.2	80	0.82
COHORT OF PATIENTS					
Arriba	19	19	86.4	100	0.93
CICERO	16	16	72.7	100	0.84
DeFuse	16	16	72.7	100	0.84
FusionCatcher	22	21	95.5	95.5	0.96
STAR-Fusion	25	20	90.5	80	0.85
Fusion InPipe 5 callers	12	12	54.5	100	0.71
Fusion InPipe 4 callers	28	18	81.8	100	0.90
Fusion InPipe 3 callers	23	22	100	95.7	0.98
GLOBAL					
Arriba	34	33	82.5	97.1	0.89
CICERO	31	30	75	96.8	0.85
DeFuse	34	28	70	82.4	0.76
FusionCatcher	44	37	92.5	84.1	0.88
STAR-Fusion	44	34	85	77.3	0.81
Fusion InPipe 5 callers	26	25	62.5	96.2	0.76
Fusion InPipe 4 callers	34	32	80	94.1	0.86
Fusion InPipe 3 callers	43	38	95	88.4	0.92

#### 3.1.2 Fusion detection in a cohort of patients’ data

We sequenced the transcriptome of 15 patients diagnosed with pediatric leukemia and previously characterized by conventional molecular methodologies. Results were analyzed using the different bioinformatics pipelines to confirm that the selected algorithms also work optimally for patients’ data.

The results obtained were similar to those achieved when analyzing cell lines’ data. The number of gene fusions called by Arriba, CICERO, deFuse, FusionCatcher, and STAR-fusion in raw data were 407, 1294, 714, 3,184, and 104 respectively. After prioritization, the total number of gene fusions retained were 48 (AR), 22 (CI), 48 (DF), 133 (FC), 25 (SF). Manual curation and visual inspection resulted in a final fusion count for each algorithm of 19, 16, 16, 22, and 25 respectively.

*STIL::TAL1* and *P2RY8::CRLF2* were the gene fusions with less percentage of detection as they were just detected by three of the five algorithms, but all the fusions were called by at least one pipeline.

For this dataset, the highest sensitivity was achieved by FusionCatcher (95.5%) followed by STAR-Fusion (90.9%), Arriba (86.4%), and CICERO (72.7%). As well as in cell lines’ analysis, deFuse presented the lowest sensitivity (72.7%). However, it achieved a precision of 100%, as well as Arriba and CICERO. The lowest precision (80%) was achieved by STAR-FUSION (see Table 1; Supplementary Figure S1).

#### 3.1.3 General performance of the individual pipelines

Bringing together all the results for the individual performance of the pipelines, a large number of artifacts were initially called [958 (AR), 2,224 (CI), 6790 (DF), 11,123 (FC), and 184 (SF)], giving a high rate of FP fusions. The use of the different filters allowed for discarding the majority of the FP fusions reducing the final number to 1, 1, 6, 7, and 10, respectively, which manifests the need for filtering steps.

**FIGURE 3:**
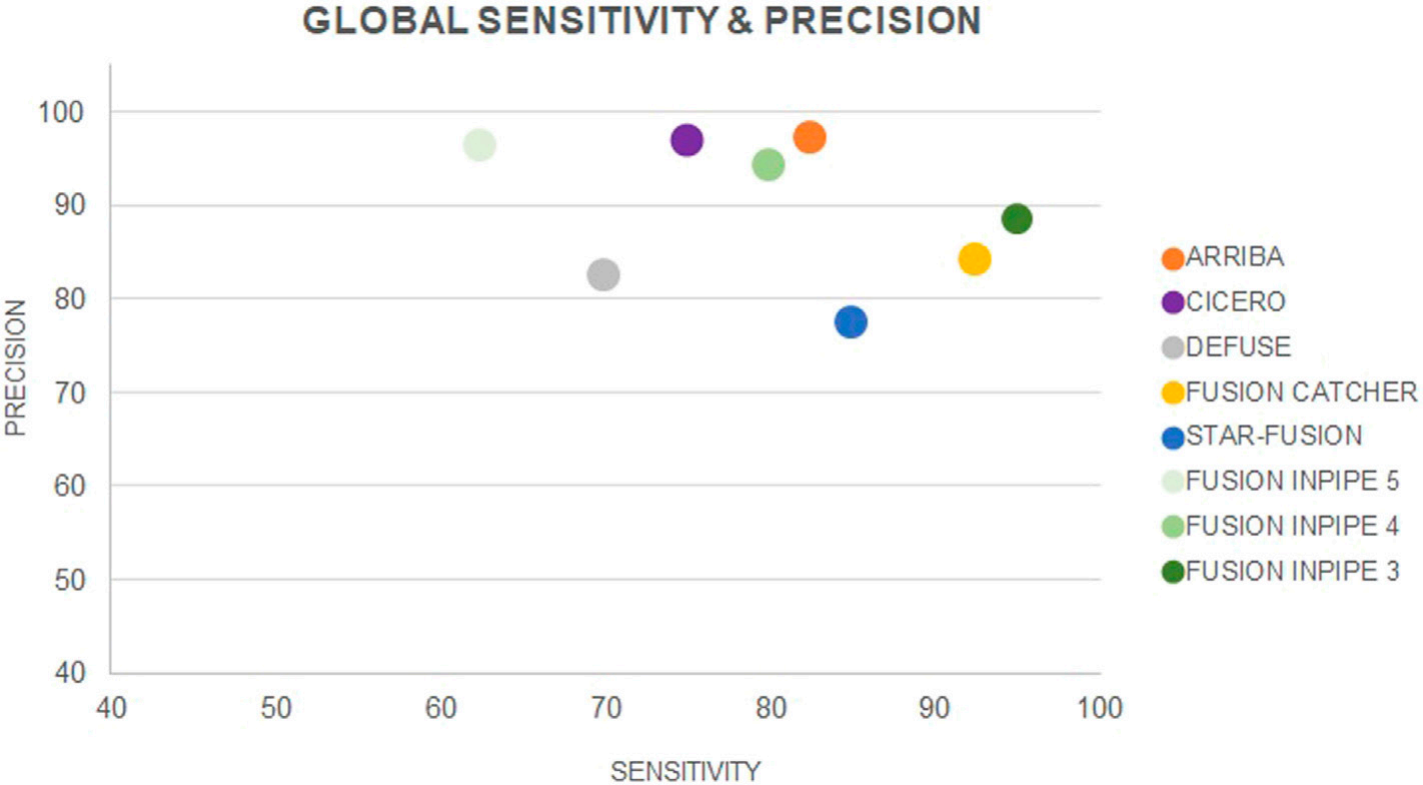
Global sensitivity and precision for each individual pipeline and for Fusion InPipe (FIP-5, FIP-4, FIP-3).

Taking into account the results obtained from the data of both the cell lines and the patients’ cohort, FusionCatcher was the pipeline with the highest sensitivity (92.5%), and Arriba had the highest precision (97.1%). On the contrary, deFuse had the lowest sensitivity (70%) and STAR-Fusion had the lowest precision (77.3%) (Figure 3 Table 1).

The F1-score showed good performance for all the pipelines working individually (range 0.76–0.89). Arriba has the better performance while deFuse has the worst (Table 1).

### 3.2 Fusion detection by Fusion InPipe

The use of a unique approach is associated with low sensitivity and poor precision. To avoid having multiple files from different tools and thus to harmonize, improve, and facilitate the analysis, we performed Fusion InPipe, which combines the output data from different pipelines: Arriba, CICERO, deFuse, FusionCatcher, and STAR-Fusion.

We compared the results obtained when we selected those fusions called by the 5 pipelines (FIP-5), by minimum 4 (FIP-4), or by minimum 3 of the pipelines (FIP-3) in both cell lines’ and patients’ data.

#### 3.2.1 Fusion detection in cell lines’ data using Fusion InPipe

For Fusion InPipe, the initial number of calls for FIP-5 was 28; we obtained 49 calls for FIP-4 and 100 for FIP-3. Once we applied our set of filters for each algorithm individually and we performed a manual curation of the fusions, the retained fusions resulted in 14 (FIP-5), 16 (FIP-4), and 20 (FIP-3).

As happened when we analyzed the data using each pipeline individually, the fusions *NKX2.5::BCL11B*, *TLX3::BCL11B*, and *LMO1::TRD* were not identified by any of the algorithms. However, the FIP-3 pipeline was able to detect the rest of the rearrangements, while FIP-4 and FIP-5 also missed other variants.

The highest sensitivity (84.2%) was achieved when we first selected the fusion genes called by a minimum of 3 pipelines (FIP-3). Despite its great sensitivity, FIP-3 presented the lowest precision (80%). On the contrary, the highest precision (92.9%) was achieved when we kept the information of the rearrangements called by all the pipelines (FIP-5), which also had the lowest sensitivity (68.4%) (Table 1; Supplementary Figure S1).

#### 3.2.2 Fusion detection in a cohort of patients’ data using Fusion InPipe

To further benchmark this approach, we tested it in our cohort. FIP-5 initially detected 23 rearrangements, FIP-4 called 43 fusions, and FIP-3 identified a total of 74. After the filtering step and the manual curation the number of called fusions was reduced to 12 (FIP-5), 18 (FIP-4), and 22 (FIP-3). Selecting those fusions called by all the pipelines, only 12 of the expected fusion genes were detected. This resulted in a sensitivity of 54.5%. When we looked at the fusions called by at least 4 algorithms the sensitivity increased to 81.8%, and only *P2RY8::CRLF2* and *STIL::TAL1* were missed. Nevertheless, all the rearrangements were called, and therefore the highest sensitivity was reached (100%) when we used data from the consensus of 3/5 pipelines (Table 1; Supplementary Figure S1).

Regarding precision, the highest value (100%) was obtained for the output derived from the consensus of the minimum of all five pipelines or at least 4 pipelines. However, the performance of 3/5 was not far from 4/5 and 5/5, presenting similar precision values (95.7%) (Table 1; Supplementary Figure S1).

#### 3.2.3 General performance of Fusion InPipe

The number of artifacts obtained for each of the options before applying the manual filters was also considerably high [26 (FIP-5), 60 (FIP-4), and 136 (FIP-3)], even though much lower than in the individual pipeline performance. However, after manually applying different filters, we discarded most of the fusions, and the number of FP retained was 1, 2, and 5 respectively.

Globally, FIP-3 was the option with the highest sensitivity (95%). The lowest sensitivity was achieved by FIP-5 (62.5%), which was the pipeline that called a less number of TP fusions. Nevertheless, FIP-5 obtained the highest precision (96.2%), and FIP-3 the lowest (88.4%) (Figure 3, Table 1). F1-score was calculated for FIP-5, FIP-4, and FIP-3 to assess the global performance of each option. All showed good results, FIP-3 being the one with the highest value (0.92) (see Table 1).

## 4 Discussion

The identification of driver fusion genes at the diagnosis is important in patients with pediatric acute leukemia, as it may help to refine the diagnosis and prognosis and be useful as a therapeutic target. Therefore, gene fusions are screened as a part of molecular pathology testing for patient management (Mertens et al., 2015). The wide range of available algorithms for the analysis of RNA-seq data and their differences in efficiency may difficult the selection of the pipeline that fits the best to the analysis. In this study, we have evaluated the performance of five of the most commonly used pipelines for the detection of gene fusions. Moreover, we have introduced Fusion InPipe, a pipeline that integrates the information of different pipelines for the identification of clinically relevant gene fusions in pediatric AL, and we have analyzed its performance.

We carried out the analysis using data from 14 different leukemia cell lines and data from a cohort of 15 previously characterized pediatric AL patients. All the pipelines separately, as well as FIP, performed well with leukemia data, as they were able to detect most of the gene fusions previously reported by gold-standard methodologies. Based on the general F1-score, Arriba was the pipeline with better individual performance (F1 = 0.89) as previously described (Uhrig et al., 2021), and FIP-3 was the option with better results using the new Fusion InPipe (F1 = 0.92).

Using only one algorithm for the identification of gene fusions can lead to poor sensitivity and precision (Carrara et al., 2013; Dupain et al., 2017). To improve this, we generated the Fusion InPipe that combines the results from different algorithms and provides the fusion candidates that are called by more than one tool. The Fusion InPipe has the advantage that is compounded by modules, so new callers can be introduced or removed at any time according to the user’s necessities. We evaluated the results obtained from the consensus calls from the five pipelines, from at least four pipelines and from at least three pipelines. As expected, trusting only those fusions called by all the pipelines employed and therefore being more restrictive resulted in low sensitivity. By contrast, when we used more permissive strategies and we selected the fusions called by three or more pipelines, the sensitivity increased and precision slightly decreased. As not all the pipelines are able to detect all the rearrangements, being too conservative may cause the loss of some TP fusions. However, it is important to avoid filters too lax to select the minimum FP fusions. Hence, it is necessary to find a balance between sensitivity and specificity such as the one obtained in our case by FIP-3.

An accurate fusion detection is important in clinical practice, as fusion detection may help to refine the diagnosis and guide targeted therapy management (Mitelman et al., 2007; Starý et al., 2018). In this sense, Fusion InPipe may be a good strategy. Each pipeline, when performed individually, resulted in different precision and recall values leading to inconsistent results between callers and consequently making difficult the analysis standardization. However, their combination using Fusion InPipe improved the accuracy, as it merged the information from all the pipelines and therefore facilitated the identification of the driver disease-causing fusion. FIP-3 presented the highest sensitivity, which demonstrates that Fusion InPipe is able to detect the highest number of TP rearrangements in contrast to the individual pipelines.

One of the biggest problems when analyzing RNA-seq data is the elevated rate of false positive fusion genes called by the pipelines (Carrara et al., 2013). Once the different pipelines are run, the generated files contain a large list of fusion candidates. Most of the fusions reported are artifacts generated by technical errors during the library generation, sequencing errors, mapping errors, etc. (Conesa et al., 2016). The initial number of called fusions differ between pipelines probably due to intrinsic characteristics of the algorithm (Supplementary Table S2). All the pipelines used for this study had the same strategy to align the reads to a reference except for deFuse, which uses bowtie instead of STAR-aligner, and Fusion Catcher which uses four different aligners including STAR-aligner and Bowtie (Supplementary Table S3). Focusing on that, we observed that the pipelines that use STAR-aligner (Arriba, CICERO, and STAR-fusion) presented an initial number of candidates lower than deFuse or Fusion Catcher which used Bowtie. Another reason that could explain differences in the initial calling is that some of the pre-filters applied can be more relaxed and therefore the initial output will contain a higher number of candidates as it happens with Fusion Catcher or deFuse. In order to determine whether a fusion is real and reduce the number of FP fusions, it is necessary to introduce filtering steps. Although each pipeline includes its own filters during the processing of the data, our study highlights the necessity of applying more filters during the analysis and performing a manual and visual curation. This is shown when after applying manual filters, the number of final candidates it is radically reduced and therefore the precision increases (Supplementary Figure S2). In the case of Fusion InPipe, the first step of selecting only the fusions called by 5, 4, and 3/5 pipelines helped to reduce the list of artifacts and FP fusions. Thus, in some cases, we excluded up to 99% of the fusions without losing any TP fusions, which is also important and demonstrates good performance of our pipelines.

In the cell lines’ training dataset, three gene fusions were not called by any of the pipelines. The non-identified fusions were present in T-ALL cases. However, none of them are currently applied to modify the clinical management of patients. In particular, *NKX2.5::BCL11B* and *TLX3::BCL11B* are two cryptic rearrangements affecting chromosomes 5 and 14. In both cases, the breakpoint in *BCL11B* covers a large region and contains base pair insertions that can difficult the alignment, which might explain the lack of detection by the algorithms (Nagel et al., 2007). *LMO1::TRD* is the third not detected rearrangement. No explanation was found to describe the lack of detection of this rearrangement other than the possible complexity of the TRD gene structure. On the other hand, *P2RY8::CRLF2* and *STIL::TAL1* were the gene fusions with a higher rate of failure to be detected by the different pipelines individually. This may be due to the biological characteristics of these rearrangements, as they are produced by intrachromosomal deletions. An explication could be that pipelines interpret these fusions as read-through fusions and therefore do not pass the internal filters to be called (Tian et al., 2020). However, we were able to detect them by our pipeline FIP-3.

Taking all of this together, this study highlights that not only more studies should be done to increase the good performance of the different pipelines to detect all the variants and not lose TP variants but also the importance of identifying those rearrangements that are difficult to detect by RNA-seq. Thus, if we suspect the presence of a fusion gene and we cannot find it using this methodology and pipelines, we should use another technique to identify or discard them.

Finally, the turn-around time is still a challenge when performing NGS methodologies. Individual fusion callers generate large final outputs that contain an extensive list of candidates. This results in an arduous and time-consuming task of prioritization that will be even larger if we want to perform the analysis with several pipelines. Fusion InPipe offers a standardized output, which facilitates the comparison of the results between the different pipelines and provides an output file that integrates all the information. Thus, only one file has to be analyzed. Taken together, it simplifies the analysis and reduces the time spent on the analysis of each patient, being able to inform the results faster.

In summary, several pipelines are currently available for RNA-seq data analysis. Although there are no standards for the analysis, it is recommended to use two or more pipelines to identify gene fusions with high confidence (Carrara et al., 2013; Mertens et al., 2015; Dupain et al., 2017). For that reason, we generated Fusion InPipe, which harmonizes and combines information from different pipelines and allows obtaining a fast and accurate gene fusion analysis. Moreover, we showed that the use of manual filters is necessary, and help to reduce FP variants and therefore, to achieve better precision.

## 5 Conclusion

Here we describe Fusion InPipe, a new bioinformatics tool that combines information from different pipelines facilitating the analysis of RNA-seq data for the detection of driver fusions in leukemia. This study reveals that Fusion InPipe is a good approach for the detection of different gene fusions involved in pediatric acute leukemia with a good balance between high sensitivity and good precision. Furthermore, we have shown that keeping those fusions called by at least 3 pipelines (FIP-3) increments the number of TP fusions detected. Altogether, the use of FIP-3 with the use of filtering steps allows us to achieve a better sensitivity performance, which is important for diagnostic analysis.

## Data Availability

The raw data supporting the conclusion of this article will be made available by the authors, without undue reservation.
