# Supplementary material for: Fusion InPipe, an integrative pipeline for gene fusion detection from RNA-seq data in acute pediatric leukemia
Source: Front Mol Biosci. 2023 Jun 9;10:1141310. doi: 10.3389/fmolb.2023.1141310 (PMC10288994; doi:10.3389/fmolb.2023.1141310)
Supplement: Supplementary file 1 [file DataSheet1.PDF]

# Supplementary Material

## 1 Supplementary Data

**Filtering steps**

Different pipelines were used for the analysis of the RNA-seq data. We applied different manual filters to reduce the number of artifacts and false positive called variants. Filters applied to each of the callers are described in the following sections:

### Arriba

1. Column "confidence": Discard low-confidence variants
2. Column "discordant_mates": Discard variants with <5 spanning reads
3. Column "split_reads1 + split_reads2": Discard variants with <3 split reads
4. Manual curation (explained below)

### CICERO

1. Column "rating": Discard rating LQ (low quality) variants
2. Column "medal": Keep variants with medal ≥3*
3. Column "readsA + readsB": Discard variants with <5 supporting reads
4. Manual curation (explained below). Moreover, have a look at column "score" (high values are supportive of true positivity).

* Estimated pathogenicity assessment using St. Jude Medal Ceremony

### deFuse

1. Column "span_count": Discard variants with <5 spanning reads
2. Column "splitr_count": Discard variants with <3 split reads
3. Column "gene_location1/gene_location2": keep those genes with the breakpoint in the coding region
4. Column "exon_boundaries": Keep Exon_boundaries=YES
5. Manual curation (explained below).

### Fusion Catcher

1. Column "Fusion_description": Discard variants with the following tags: Bodymap2, ribosomal, Banned, Pseudogene, lncRNA.
2. Column "Spanning_pairs reads": Discard variants with <5 spanning reads
3. Column "Spanning_unique_reads": Discard variants with <3 split reads

4. Manual curation (explained below). Moreover, review the “Fusion_finding_method” column and keep variants found with more than one aligner (BOWTIE/BLAT/STAR/BOWTIE2).

**STAR-Fusion**

1. Column “SpanningFrag”: Discard variants with <5 spanning reads
2. Column “JunctionReads”: Discard variants with <3 split reads
3. Manual curation (explained in the main text).

## 2 Supplementary Figures and Tables

### 2.1 Supplementary Figures

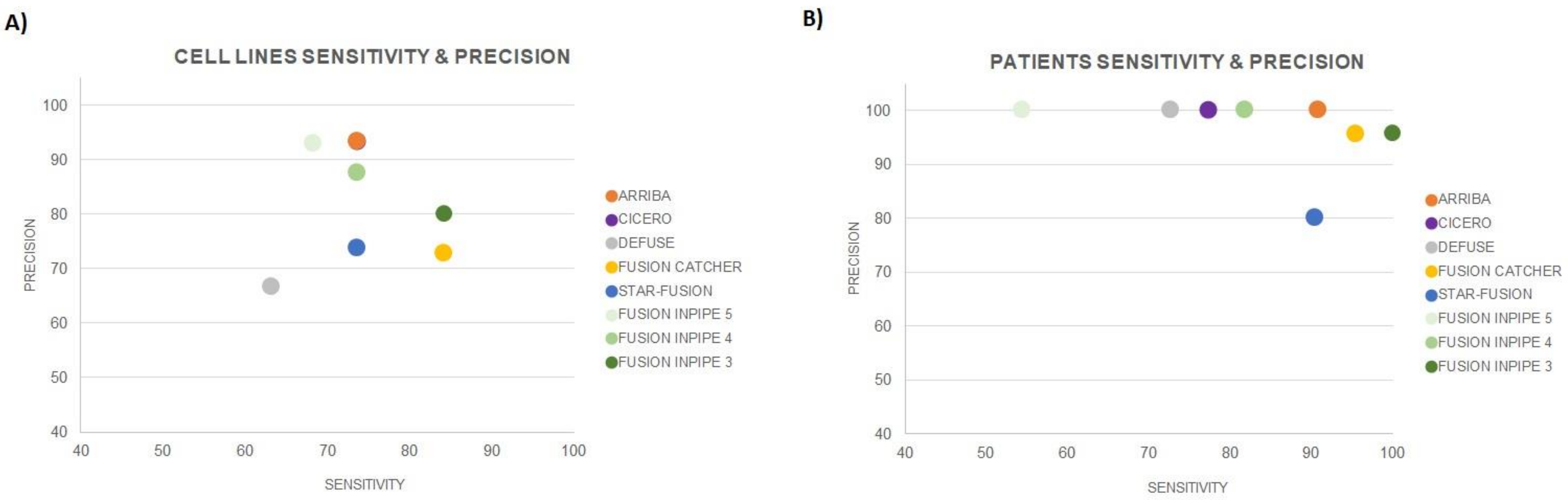


**Supplementary figure 1.** Sensitivity and precision graphics obtained by the different pipelines for a) cell lines and b) patients.

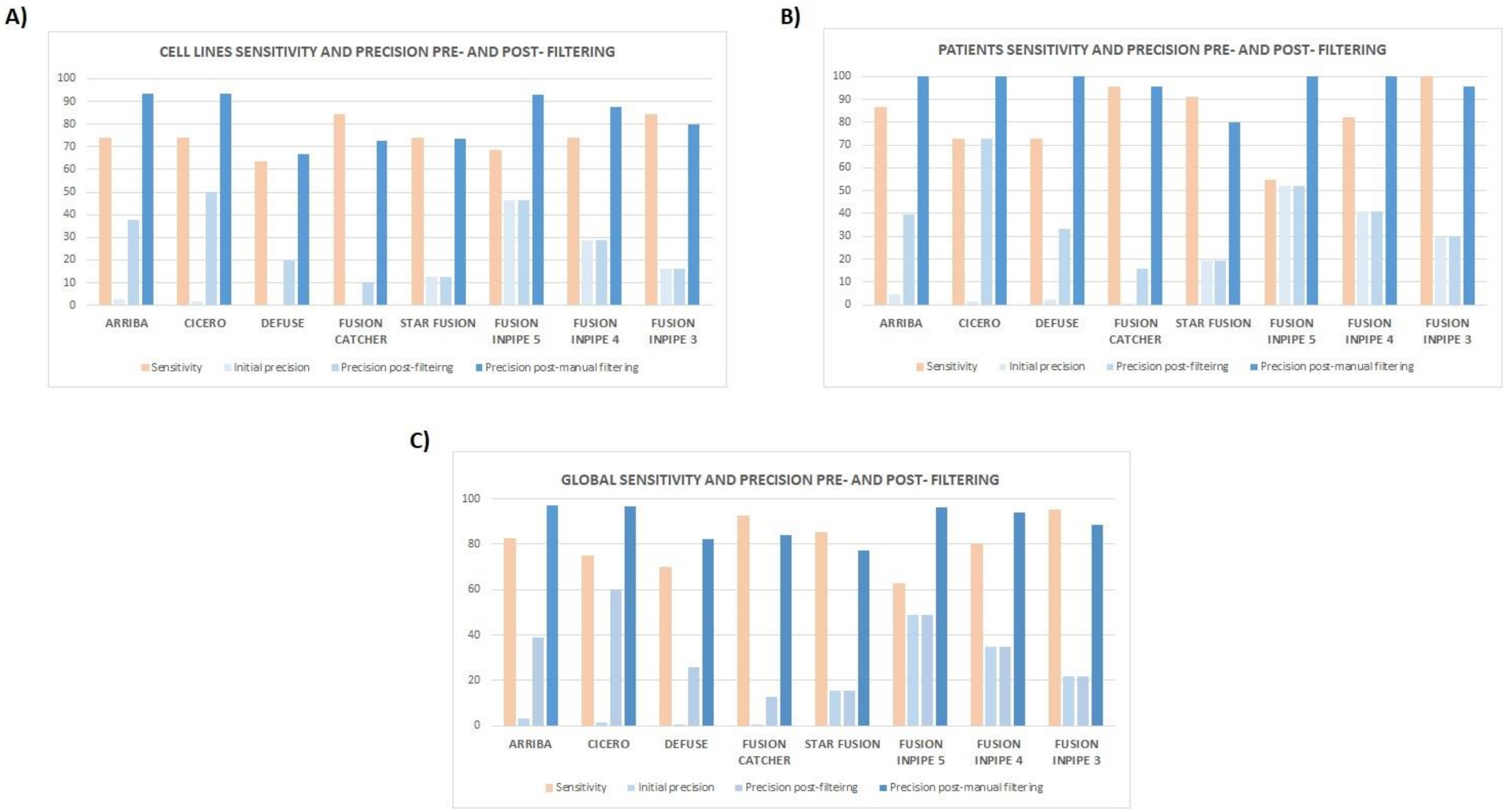


**Supplementary figure 2.** Differences in precision before and after applying the filters for each pipeline. Orange bars show sensitivity. Data is shown separately for A) Cell lines, B) Patients, and C) Global.

## 2.2 Supplementary tables

| Cell line | Leukemia subtype | Age/Gender | Alteration | |
|---|---|---|---|---|
| **EOL-1 (ERR3003531)** | acute myeloid leukemia | 33 year/male | *FIP1L1::PDGFRA* | del(4)(q12q12) |
| **HL-60 (ERR3003540)** | acute myeloid leukemia | 36 year/female | *CYFIP2::PLCG2* | t(5;16)(q33.3;q23.3) |
| **KASUMI-1 (ERR3003548)** | acute myeloid leukemia | 7 year/male | *RUNX1::RUNX1T1* | t(8;21)(q22;q22) |
| **KG-1 (ERR3003550)** | acute myeloid leukemia | 59 year/male | *FGFR1OP2::FGFR1* | t(8;12)(p12;p11) |
| **NB-4 (ERR3003575)** | acute myeloid leukemia | 23 year/female | *PML::RARA* | t(15;17)(q22;q12) |
| **SKNO-1 (ERR3003594)** | acute myeloid leukemia | 22 year/male | *RUNX1::RUNX1T1* | t(8;21)(q22;q22) |
| **697 (ERR3003513)** | B-Cell Precursor-acute lymphoblastic leukemia | 12 year/male | *TCF3::PBX1* | t(1;19)(q23;p13) |
| **KOPN-8 (ERR3003554)** | B-Cell Precursor-acute lymphoblastic leukemia | 3 month/female | *KMT2A::MLLT1* | t(11;19)(q23;p13.3) |
| **REH (ERR3003588)** | B-Cell Precursor-acute lymphoblastic leukemia | 15 year/female | *ETV6::RUNX1* | t(12;21)(p13;q22) |
| | | | *RUNX1::PRDM7* | t(16;21)(q22;q16) |
| **SEM (ERR3003592)** | B-Cell Precursor-acute lymphoblastic leukemia | 5 year/female | *KMT2A::AFF1* | t(4;11)(q21;q23) |
| **CCRF-CEM (ERR3003519)** | T-cell acute lymphoblastic leukemia | 4 year/female | *NKX2.5::BCL11B* | t(5;14)(q35;q32.2) |
| **DND-41 (ERR3003527)** | T-cell acute lymphoblastic leukemia | 13 year/male | *TLX3::BCL11B* | t(5;14)(q35;q32.2) |
| **HPB-ALL (ERR3003541)** | T-cell acute lymphoblastic leukemia | 14 year/male | *CBFB::MYLPF* | inv(16)(p13q22) / t(16;16)(p13;q22) |
| **RPMI-8402 (ERR3003591)** | T-cell acute lymphoblastic leukemia | 16 year/female | *LMO1::TRD* | t(11;14)(p15;q11) |
| | | | *SIL::TAL1* | del(1p32) |

**Supplementary Table 1.** Main characteristics of the different leukemia cell lines used for the analysis.

| **Sample** | **Sex** | **Leukemia subtype** | **Karyotype** | **FISH** | **RT-qPCR** |
|---|---|---|---|---|---|
| Sample 1 | Female | B-ALL | 46,XX,?t(7;9),?t(9;12),t(9;22)(q34;q11)(22)/46,XX(3) | 97% of the nuclei with *BCR::ABL1* rearranged | *BCR::ABL (P210)* |
| Sample 2 | Male | B-ALL | 47, XY,t(9;22)(q34;q11),+17 | 87% of the nuclei with *BCR::ABL1* rearranged | *BCR::ABL (P190)* |
| Sample 3 | Male | B-ALL | 46,XY,del(6)(q?13q?23),add(9)(q34),del(12)(p13), del(13)(q?12q?14)(9)/47,idem,+?10(8)/46,XY(3) | Not performed | *ETV6::RUNX1* |
| Sample 4 | Male | B-ALL | 46,XY,del(6)(q13q25)[4]/46,XY[34] | 50% of the nuclei with *ETV6::RUNX1* rearranged, 35% of them with deleted *ETV6* | *ETV6::RUNX1*<br>*P2RY8::CRLF2* |
| Sample 5 | Female | B-ALL | 46,XX,der(19)t(1;19)(q23;p13)[6]/46,XX[46] | 27% of the nuclei with *TCF3* rearranged. | *TCF3::PBX1* |
| Sample 6 | Female | B-ALL | 46, XX | 98% of the nuclei with *KMT2A* rearranged | *KMT2A::AF4* |
| Sample 7 | Male | AML | 46,XY,der(11)(q23)[3]/46,XY[17] | 22% of the nuclei with *KMT2A* rearranged | *KMT2A::AF9* |
| Sample 8 | Male | T-ALL | no metaphases | No alterations identified | *STIL::TAL1* |
| Sample 9 | Male | T-ALL | 46,XY[5] | No alterations identified | *STIL::TAL1* |

| | | | | | |
|---|---|---|---|---|---|
| Sample 10 | Female | AML | 47,XX,+8[3]/47,XX,+1[2]/46,XX[5] | Not performed | *RUNX1::CBF2AT3* |
| Sample 11 | Male | B-ALL | no metaphases | 86% of the nuclei presented a monoallelic deletion of *JAK2* | *PAX5::NOL4L* |
| Sample 12 | Male | AML | 47, XY, del(7)(q?32), inv(16)(p13q22), +22 [17] | Not performed | *CBFB::MYH11* |
| Sample 13 | Female | AML | 46,XX,t(15;17)(q24;q21) | Not performed | *PML::RARA* |
| Sample 14 | Male | B-ALL | 47,XY,+5[10]/46,XY[8] | 97% of the nuclei presented signs suggestive of chromosome 5 trisomy | *P2RY8::CRLF2* |
| Sample 15 | Female | B-ALL | 46,XX,del(5)(q33),del(9)(p22),-12,-20,+mar1,+mar2[26] / 46,XX[7] | 28% of the nuclei presented 3 copies (2 smaller) of *ETV6*, suggesting a rearrangement. 60% of the nuclei presented 3 copies (2 smaller) of *ABL1*, suggesting a rearrangement. | *ETV6::ABL1* |

**Supplementary Table 2.** Main alterations identified by different conventional methodologies in patients used for the sequencing and the analysis of the pipelines.

| Pipeline | Workflow | Aligner | Fusion calling based on | Filters | Reference |
|---|---|---|---|---|---|
| **Arriba** | 1- Alignment<br>2- Detection of chimeric reads<br>3- Filtering step | STAR-aligner | Split reads<br>Discordant reads | Read-level (single read)<br>event-level (multiple reads) | Uhrig et al., 2021<br>Genome Res |
| **CICERO** | 1- Alignment<br>2- Identification of fusion breakpoint<br>3- Fusion annotation<br>4- Ranking of fusion candidates (filtering) | STAR-aligner | Discordant reads<br>Soft clipped reads | signal-to-noise-recognition procedures | Tian et al., 2020<br>Genome Biol |
| **deFuse** | 1- Alignment<br>2- Build a list of candidates<br>3- AdaBoost classifying (filtering) | Bowtie | Discordant reads<br>Split reads | dynamic programming<br>AdaBoost classifier | McPherson A et al., 2011<br>PLoS Comput.Biol |
| **Fusion Catcher** | 1- Alignment (4 methods)<br>2- Building of a preliminary list of candidates<br>3- Filtering step | Bowtie, BLAT, STAR-aligner, Bowtie2 | Split reads<br>Discordant reads | ChimerDB2, CACG, and ConjoinG<br>BodyMap2<br>ad-hoc/random fusion | Nicorici et al., 2014<br>bioRxiv |
| **STAR-fusion** | 1- Alignment<br>2- Fusion annotation<br>**3-** Filtering step | STAR-aligner | Split reads<br>Discordant reads | FusionFilter framework | Haas et al., 2019<br>Genome Biol |

**Supplementary table 3.** Main characteristics of the different algorithms analyzed.
